# Real-Time Motion Analysis System Using Low-Cost Web Cameras and Wearable Skin Markers

**DOI:** 10.3389/fbioe.2021.790764

**Published:** 2022-01-17

**Authors:** Kun-Do Lee, Hyung-Soon Park

**Affiliations:** Neuro-Rehabilitation Engineering Laboratory, Korea Advanced Institute of Science and Technology, Dept. Mechanical Engineering, Daejeon, South Korea

**Keywords:** wearable skin marker, home rehabilitation, image motion analysis, motion capture, image processing

## Abstract

COVID-19 has restricted outdoor exercise and hospital visits for rehabilitation therapy. Home-based training and rehabilitation coaching systems have emerged as a way to overcome these circumstances. Conventional optical motion-capture systems, such as VICON, have been used for measuring precise movement and providing posture feedback during exercise or rehabilitation; however, its application is limited to professional facilities because of its high cost and space requirement. To extend the applicability to home-based use, we designed wearable skin markers (WSMs) with body segment-specific patterns that can be detected by low-cost web cameras. WSMs are band-shaped and stretchable and thus can be worn like cloth, with minimal effort for placement. The body segment-specific patterns enable real-time data processing, which reduces the marker data post-processing time. A 6-degree-of-freedom (DOF) pose for each WSM is obtained by recognizing the segment-specific patterns; the 3D configuration of the contoured corners of the patterns found by triangulation is then utilized to construct the coordinates of each WSM. The WSM system was validated *via* three experiments. The robustness of marker recognition was evaluated by measuring the false-positive and false-negative rates of WSM. For accuracy validation, the angle estimation results were obtained for the mechanical joint of a 3-DOF gimbal and lower-limb joints of a walking human subject and compared to the reference systems. The gimbal experiment was included to evaluate the accuracy of our system in the condition with no skin movement artifact. The maximum standard deviation of the difference between WSM and the encoder was 0.9 °  for the gimbal experiment, and that between WSM and VICON was 5.0 ° for the human experiment. The accuracy was comparable to the reference systems, making it suitable for home environment application.

## 1 Introduction

OPTICAL motion-capture systems that include high-end infrared cameras, such as VICON (Vicon Motion Systems, Oxford, United Kingdom) and OptiTrack (NaturalPoint, Inc., OR, United States) systems, are considered to be gold-standard systems for motion-analysis research. They are employed in biomechanics laboratories and in large hospitals that conduct studies on the pathological movement patterns of patients. The kinematic data acquired from these systems are applied in medical and sports applications to analyze and evaluate the quality of human movement. These systems can also be used in domains such as robotic research and the film industry. However, the applicability of these systems is limited by their high cost and the technical skills required to collect and analyze data.

The protracted COVID-19 pandemic has highlighted the need for home-based training and rehabilitation systems. Such a system would require that motion-capture systems be designed for home-based use, as they play an important role in pose feedback during exercise. The ideal motion-capture system in a home environment should not only be able to provide accurate pose estimation but also be affordable and simple enough to be used by individuals who are unfamiliar with the motion-capture technique. Several low-cost motion-capture systems are introduced in the following paragraphs.

One of the most widely used low-cost motion-analysis systems utilizes inertial measurement units (IMUs) composed of accelerometers and gyroscopes that provide information about body position and orientation. In the case of gait analysis, the angular kinematics (e.g., pelvis tilt and hip flexion/extension) and spatiotemporal gait parameters (e.g., step length and speed) can be analyzed by attaching IMUs to different segments of the lower half of the body, such as the pelvis, thigh, or shank. Unlike optical motion-capture systems, IMU-based motion-analysis systems must integrate the raw data to obtain the absolute pose of the segments. However, the integrated sensor noise contributes to the steadily reduced positional and rotational data accuracy over time (Brennan et al., 2011; Seaman and McPhee, 2012; Filippeschi et al., 2017; Zhang et al., 2017). Brennan et al. determined the maximum angular drift of an IMU on a 3-degree-of-freedom (DOF) gimbal to be 1°/min; they reported it to considerably affect joint-angle estimation accuracy (Brennan et al., 2011). Thus, marker-based optical motion-capture systems are better equipped than IMU-based systems to accurately determine the absolute position and orientation of body segments.

There are markerless motion-capture systems that can be used with low-cost cameras. Convolutional neural network-based motion-capture systems, such as OpenPose, have been developed. Implementation of these systems with multiple cameras allows the positions of the body joints in 3D space to be obtained via triangulation (Cao et al., 2017; Slembrouck et al., 2020). Kinect sensors (Microsoft Corp., NM, United States) have also been used to determine joint location without the use of body markers (Pfister et al., 2014; Bilesan et al., 2019). Although these types of systems are convenient, in that the users are not required to wear any markers, because they only provide joint location data, it is difficult to determine the joint rotation angle in the transverse plane (e.g., forearm supination/pronation and internal/external rotation of shoulder/knee/hip joints) (Bilesan et al., 2019). There is another type of motion-capture system that extracts the human body silhouette from captured images and then fits the 3D human skeletal model to obtain the kinematic parameters (Corazza et al., 2006; Becker and Russ, 2015). However, this method is also inadequate in terms of its ability to determine the joint rotation angle in the transverse plane because the changes in the silhouette during rotation are not sufficiently large to enable precise motion tracking.

Some researchers have utilized augmented reality (AR) markers to track specific movements (Mostashiri et al., 2018; Nagymáté and Kiss, 2019). Nagymáté et al. developed an affordable AR marker-based gait-analysis system (Nagymáté and Kiss, 2019). A total of five AR markers were attached to the pelvis, thighs, and shanks, and the locations of the virtual anatomical points were calibrated relative to the AR markers. The locations of the virtual anatomical points and orientations of the segments were obtained by tracking the poses of the AR markers. Their approach only requires one camera to determine the AR marker poses. However, accurate pose estimation requires that the AR markers be sufficiently large and composed of a rigid material, creating conditions that may negatively impact the gait of patients with walking disorders.

In this study, we propose wearable skin markers (WSMs) that can be recognized by ordinary RGB cameras for low-cost and accurate motion analysis. The WSM pattern was printed on stretchable span fabric. To ensure comprehensive evaluation of the WSM-based system, the robustness of WSM recognition was assessed by false-positive (FP) and false-negative (FN) analyses, and two types of experiments were conducted for pose estimation accuracy evaluation using a 3-DOF gimbal and with a healthy human subject.

The primary purpose of this study was to introduce a new method for low-cost optical motion analysis. Specifically, the WSM concept was designed in this study to enable 6-DOF kinematic analysis of body segments. WSMs can be detected by low-cost web cameras, thereby reducing the cost of motion-capture systems. Moreover, they can be stretched according to the size of body segments, allowing them to be worn like cloth to reduce preparation time. The second aim was to evaluate the accuracy of the proposed system by comparing to well-known measurement systems (e.g., digital encoder and a professional optical motion-capture system).

## 2 Methods

### 2.1 Wearable Skin Marker Design

#### 2.1.1 Overall Design of Wearable Skin Markers

**FIGURE 1:**
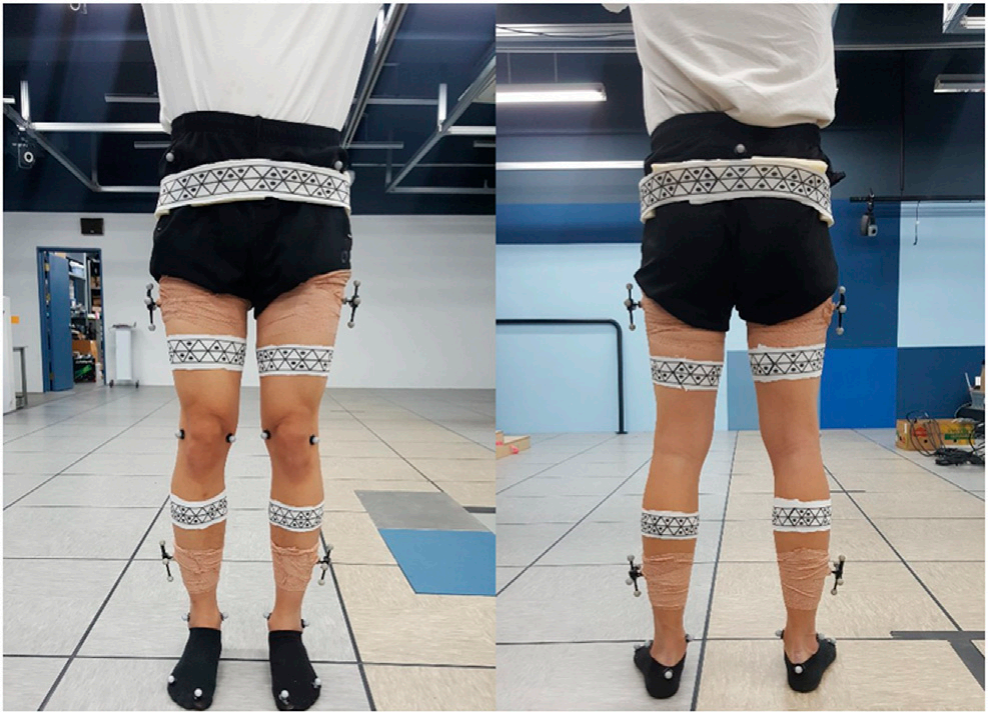
Marker positioning. WSMs and retroreflective markers were positioned on the lower limb to evaluate the joint-angle estimation accuracy of the WSM system.

Figure 1 shows the human subject wearing WSMs and the retroreflective markers for the experiment. WSMs were made in the shape of a skinny band to ensure that they would not impede natural movement and to widen the area for camera locations where the markers are visible. The material is span fabric, allowing it to stretch and shrink according to the size of the subject’s body parts. On WSM, a continuous pattern of triangles with or without the black dots inside is printed. Each WSM has its own body-segment-specific pattern so that it allows the cameras to recognize and distinguish each WSM. When two or more cameras detect the same pattern of a WSM, the 3D configuration of triangle vertices that compose the WSM can be obtained. Vertex configuration is used to determine the body segment poses defined relative to it. Thus, even when all of the vertex positions are not detected during motion analysis, multilateration and the least squares method can be used to estimate the 6-DOF pose of each body segment. The detailed procedure for pose estimation is described in sections 2.2 and 2.3, and the following paragraphs describe the detailed design decision process of WSM.

#### 2.1.2 Adopting Equilateral Triangle as a Basic Polygon to Compose the Pattern of Wearable Skin Markers

The Ramer–Douglas–Peucker (RDP) algorithm, which approximates contours as polygons by specifying the locations of vertices, was implemented for polygon recognition to enable robust pattern recognition, even under the conditions of curved surfaces that result in distortion of the marker pattern in the image (Saalfeld, 1999). If the internal angle of polygons that compose the marker pattern is large, the vertex might be missed during the line simplification procedure of the RDP algorithm. Therefore, each pattern was designed with equilateral triangles as smaller internal angles help robust recognition of vertices in the RDP algorithm in the presence of marker pattern distortion. The triangles were arranged to meet at a point to form hexagons, and the continuous pattern covered the marker surface.

#### 2.1.3 Required Number of Pattern IDs in Wearable Skin Markers

**FIGURE 2:**
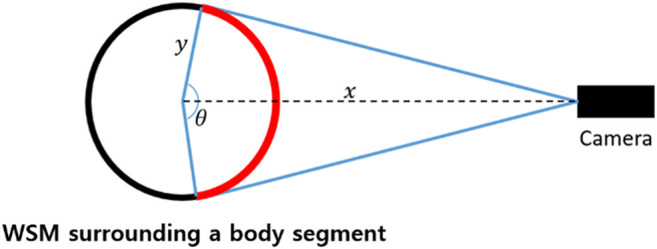
The number of pattern IDs needed for a WSM was decided by considering that at least two pattern IDs should be visible in the red region, thereby ensuring that at least one pattern ID can be recognized from any direction.

To obtain the vertex configurations of a WSM, multiple patterns should be employed, each with their own ID, as this would allow each WSM to be individually recognized by cameras at different locations. Regarding the process of determining a suitable number of pattern IDs for a single WSM, we assumed that at least two IDs should be able to fit in the red region shown in Figure 2 to ensure that at least one ID would be able to be recognized by each camera. The red region denotes the area on a WSM that is visible by a camera. To determine *θ*, which reflects the range of the red region, the following three additional assumptions were made: (1) the transverse section of each body segment is circular, (2) the marker is at least 1 m away from all cameras (i.e., *x* is larger than 1 m), and (3) the radius of any body segment *y* is less than 23 cm, as obtained from the results of an anthropometric survey of United States Army personnel (Gordon et al., 1989). Thus, the smallest possible *θ* is 153°. Additionally, to ensure reliability, when at least two IDs were able to fit within a 120° range, each WSM was required to have six IDs.

#### 2.1.4 Definition of Pattern ID

**FIGURE 3:**
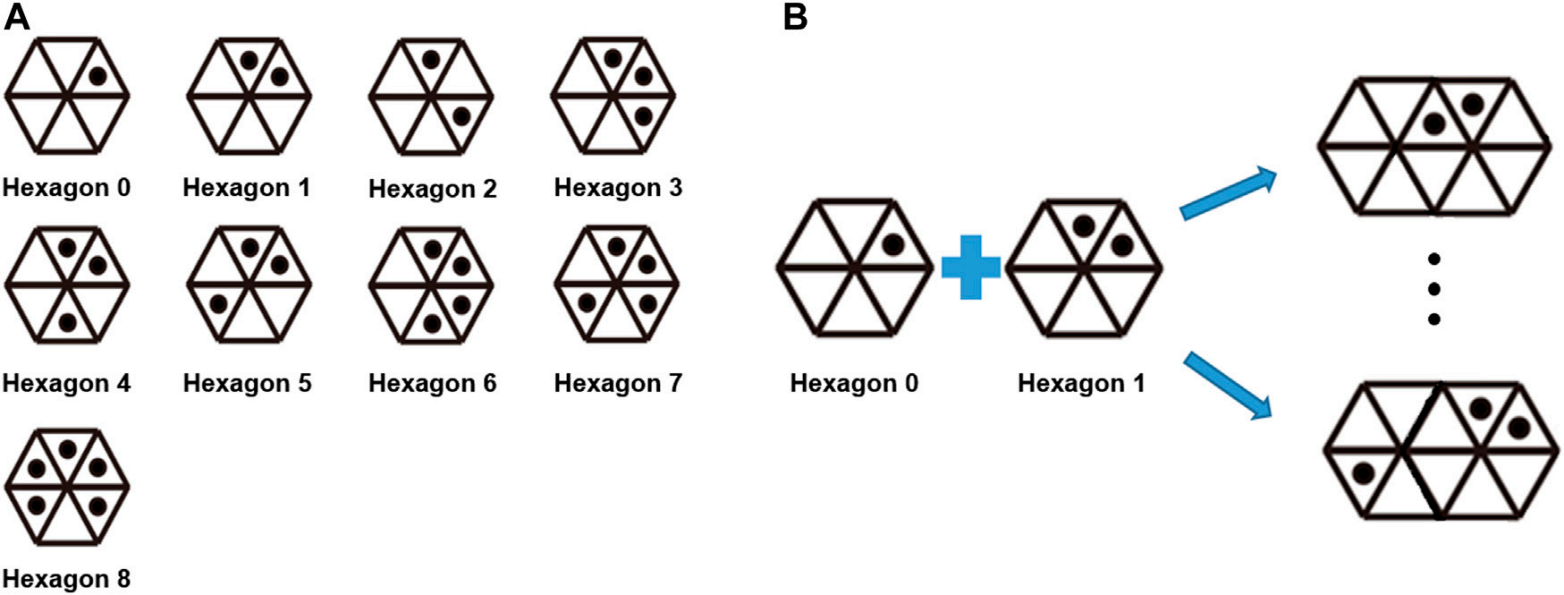
**(A)** Pattern IDs implemented in the WSM system. Note that radially symmetric patterns were avoided within a single hexagon. **(B)** A total of 384 pattern IDs can be used when two adjacent hexagons are used to compose an ID.

We first considered the hexagonal patterns shown in Figure 3A. These patterns can be identified by decoding clockwise the sequence of triangles with or without the interiorly positioned black dots. To ensure that each triangle making up each hexagon could be distinguished, radially symmetric patterns were avoided. As such, nine hexagonal patterns were found to be possible (Figure 3A). However, this number is insufficient when there is more than one body segment to be analyzed. Therefore, we decided to use two neighboring hexagons to define an ID (Figure 3B). Consequently, a total of 384 pattern IDs were found to be possible following analysis of the patterns of single hexagons and all of the patterns that can be generated by merging and rotating neighboring hexagons in 60° increments. Note that the number of pattern IDs could be increased by adding the number of dots in a single triangle, instead of merging two neighboring hexagonal patterns; however, this could lead to marker ID confusion when the dots overlap in the image because of the pattern distortion in the image caused by the curved surface.

#### 2.1.5 Size of Triangles Required for Robust Wearable Skin Marker Pattern Recognition

The camera resolution and distance between the cameras and subject of motion analysis should be considered when deciding the size of the pattern triangles. According to the theory behind the pinhole camera model, the relationship between the actual size and pixel size of the triangles can be described as

$$\frac{A}{T^{2}}=\frac{A_{pixel}}{f_{x}f_{y}}$$

where *A* and *A*_*pixel*_ are the actual area and pixel area of the triangles, respectively, and *T* is the actual distance between the focal center of the camera and the pattern triangle. *f*_*x*_ and *f*_*y*_ are the horizontal and vertical focal lengths of the camera, respectively, in pixels. The size of the triangle was decided after determining *T* and the smallest triangle pixel area that would allow for robust recognition. Thus, an experiment was performed to confirm the smallest pixel area of each triangle required for robust recognition of WSM; the results revealed that an area larger than 80 pixels was required. The detailed experiment is described in sections 3.1 and 4.1. The focal length parameters for the camera were determined by using the “calibrateCamera” function of OpenCV and a chessboard pattern plate. Consequently, *f*_*x*_ and *f*_*y*_ were calculated to be 1,452.59 and 1,439.01, respectively. The cameras around the treadmill were positioned to be a maximum of 2 m from the WSMs on the moving subject. Thus, the triangle area was required to be at least 1.53 cm^2^. In this study, equilateral triangles, each with a side length of 2 cm and an area of 1.73 cm^2^, were used to make up the patterns.

### 2.2 Marker Recognition Algorithm

**FIGURE 4:**
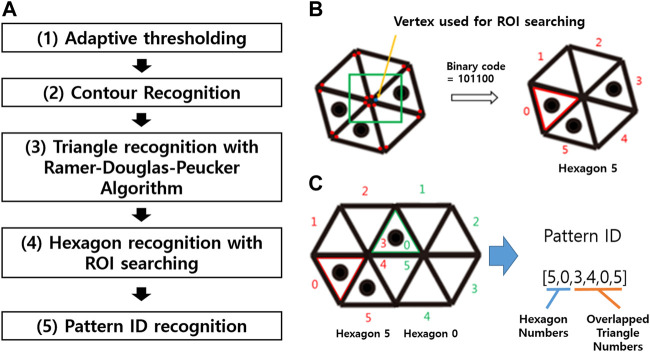
**(A)** Flow chart summarizing the steps to pattern recognition. (1) Binarize the original RGB images using an adaptive thresholding method. (2) Recognize the boundaries of binarized images to obtain the contours. (3) Use the Ramer–Douglas–Peucker algorithm to identify the triangles and vertex locations from the contours. (4) Identify the hexagonal patterns by decoding the binary code. (5) Recognize the pattern ID by determining which and how the neighboring hexagons are integrated. **(B)** Individual hexagonal pattern identification process. Using the square ROI centered about the triangle vertices found in Step 3, when six vertices are included in the ROI, the binary code for the triangles is read clockwise, beginning with the vertex corresponding to the ROI center. The binary code is used to recognize the hexagonal patterns and each corresponding triangle, because the hexagonal patterns are not radially symmetric. **(C)** Integrated hexagonal pattern identification process. The final pattern ID can be recognized by identifying the overlapping triangles. Here, Triangles 3 and 4 of Hexagon 5 are overlapping with 0 and 5 of Hexagon 0.

As described by the flow chart shown in Figure 4A, the marker recognition process consists of five steps. In Step 1, the adaptive thresholding method is used to create binary images. The processes of contour recognition and triangle recognition are respectively performed in Steps 2 and 3 based on the binary images. In Step 4, the binary code for the hexagon and a region-of-interest (ROI) searching technique are used to identify the individual hexagonal pattern (Figure 3A). Specifically, a square ROI that is centered at the vertex and has the same side length as the corresponding triangle is defined for each triangle vertex. When the ROI contains six vertices, the binary code for the hexagon is scanned clockwise, beginning with the triangle associated with the ROI center (Figure 4B). This binary code is then utilized to identify the individual hexagonal pattern and, consequently, the comprising triangles. Lastly, in Step 5, the overlapping triangles of the neighboring hexagons are identified, and the final pattern ID is determined (Figure 4C). When two or more cameras output the same ID, the 3D locations of the vertices of the pattern can be determined by triangulation.

### 2.3 Pose Estimation for Wearable Skin Markers

#### 2.3.1 Static Step

**FIGURE 5:**
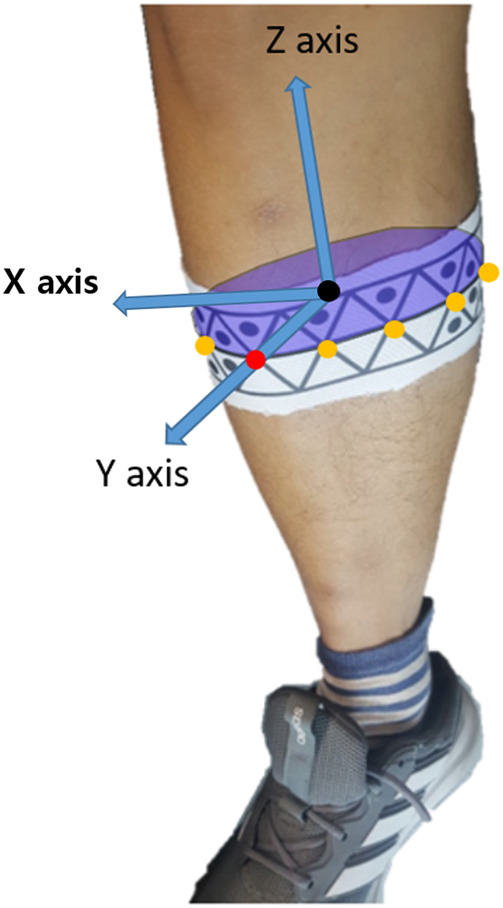
Example of the pose estimation referencing system. Each body segment was defined with respect to the anterior (positive *Y* axis) and proximal (positive *Z* axis) directions. The positive X axis corresponds to the cross product of *Y* and *Z* axes.

**FIGURE 6:**
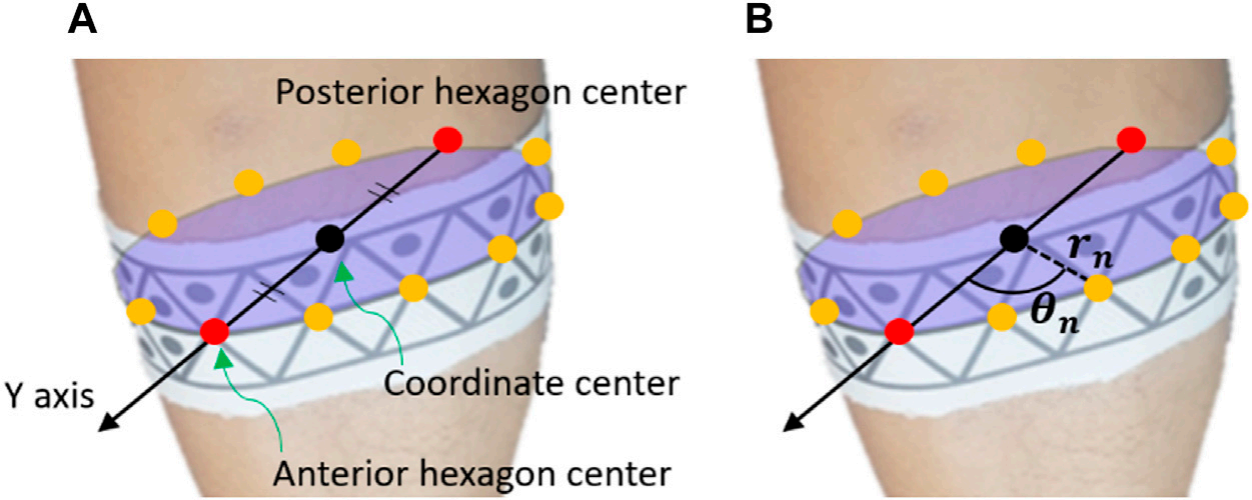
Illustrative example of how the pose estimation referencing system was utilized to determine the geometric parameters of a body segment. In the static step, the geometric parameters of each body segment are collected. As shown in **(A)**, the positive *Y* axis corresponded to the anterior direction of body segments; it was defined as the vector extending from the center of the posterior hexagon to the center of the anterior hexagon. These centers were predefined as the respective centers of the anterior and posterior points of each body segment and were thus positioned as such. The origin was defined as the midpoint between the centers of the posterior and anterior hexagons. As shown in **(B)**, the distance between the origin and each vertex of each marker (i.e., not only the hexagon centers) was denoted as *r*_*n*_. Additionally, the angles between the positive *Y* axis and the vectors for each hexagon center were determined (*θ*_*n*_). *r*_*n*_ and *θ*_*n*_ were utilized in the motion-analysis step to reconstruct the positive *Y* axis and origin when the anterior and posterior hexagons could not be recognized.

The pose estimation process comprised two steps: the static step and motion-analysis step. In the static step, the geometric parameters of the body segments with applied WSMs were calculated as the user stood still in the predetermined area for motion analysis. As shown in Figure 5, the positive *Y* axis of the body segments corresponded to the anterior direction, and the positive *Z* axis corresponded to the proximal direction. Figure 6 provides insight into the static step, showing how the geometric parameters were determined relative to the pose of the WSM on a body segment. For each WSM, there was one hexagonal pattern that was predefined as the anterior or posterior part of each body segment, and the center of each of these hexagons was positioned as the center of the corresponding anterior or posterior body segment. The positive *Y* axis has been defined as the vector extending from the center of the posterior hexagon to the center of the anterior hexagon. The positive *Z* axis has been defined as the normal vector of the plane fitted to the center of all hexagons on each WSM; the origin of the coordinate system has been defined as the midpoint between the centers of the posterior and anterior hexagons.

After the coordinates were defined, the distance from the origin was calculated for each WSM vertex. Then, in the motion-analysis step, these data were implemented in a multilateration process to estimate the origin. Multilateration is the process of determining the position of a point in 3D space by measuring and referencing its relative distances from more than two known points (Blewitt, 1997). The angles between the *Y* axis and the vector from origin to each of the hexagon centers are also calculated in the static step. These angles were used to find the *Y* axis in the motion-analysis step when the posterior or anterior hexagon was not recognized.

#### 2.3.2 Motion-Analysis Step

It should be noted that, because the motion-analysis step does not ensure that the posterior and anterior hexagons can be recognized by cameras while the subject is in motion, the origin and *Y* axis were estimated based on the information obtained in the static step. To summarize the process of the motion-analysis step, first, the position of the origin is determined by using multilateration based on the location of vertices of the WSM currently found. Then, by fitting the plane to the centers of identified hexagons and the estimated origin, the normal vector of the plane is established as the positive *Z* axis. Lastly, the *Y* axis is identified in the motion-analysis step by rotating the vector extending from one of the identified hexagon centers about the *Z* axis according to the aforementioned angles determined in the static step.

## 3 Experiment Design for System Validation

### 3.1 Experiment 1: Robustness of Marker Recognition

We analyzed the FP and FN rates of WSM to evaluate its robustness of recognition. The FP rate is the proportion of falsely detected markers in the LabelMe dataset (Russell et al., 2008). LabelMe is composed of 207,913 images of various indoor and outdoor scenes that do not contain WSM images.

The FN rate is the proportion of undetected WSMs when there are WSMs in the image. We attached a WSM around a cylinder with 7 cm diameter. We fixed the size of the marker, and the length of one side of the equilateral triangles constituting the pattern was 2 cm. We obtained the minimum pixel area of a WSM for robust marker recognition adjusting the distance between WSM and the camera.

### 3.2 Experiment 2: Joint-Angle Estimation for a 3-DOF Gimbal

**FIGURE 7:**
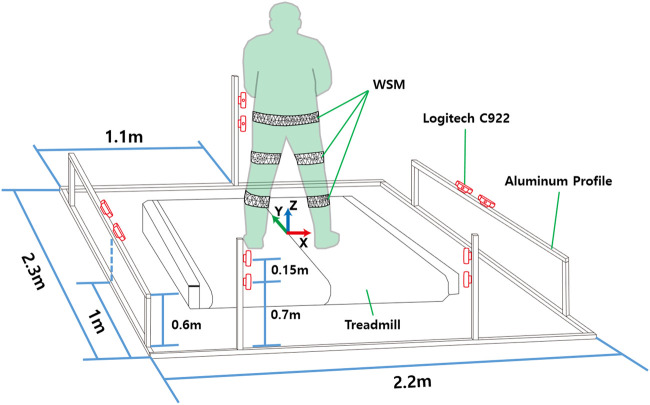
Camera locations for 3-DOF gimbal and human subject experiments. The world coordinates were defined on the center of the treadmill.

For Experiments 2 and 3, the motion-capture area was surrounded by 10 Logitech C922 webcams (68 USD each) for the WSM system as shown in Figure 7 and eight infrared cameras (i.e., camera types: five VICON T40-S, two T160, and one T040) for the VICON-based system. The webcams were placed in pairs for correspondence-based triangulation. Five pairs of webcams were placed as shown in Figure 7.

To evaluate system accuracy, joint-angle estimation tests were conducted using a 3-DOF gimbal and on the lower body of a healthy subject. A 3-DOF gimbal test was included to eliminate the effects of skin movement artifacts, which consequently reduce the accuracy of marker-based motion-analysis systems (Ferrari et al., 2008; Gao and Zheng, 2008). Gao and Zheng (2008) conducted a study in which 20 healthy subjects performed a walking task with retroreflective marker clusters attached to the same body segment with different locations; they consequently found that skin movement caused maximum discrepancy between the marker clusters on the thighs and shanks approximately 19.6° and 8.6° in the transverse plane, 12.0° and 2.6° in the sagittal plane, and 5.9° and 2.7° in the frontal plane. Because, in this study, the WSMs and retroreflective markers needed to be positioned on the same body segment, but at different locations to prevent obstruction, the results of the human subject experiment were presumed to be susceptible to skin movement artifacts. Thus, a gimbal experiment was performed to enable comparison of the proposed system to a VICON-based system under the condition of no skin movement artifacts.

**FIGURE 8:**
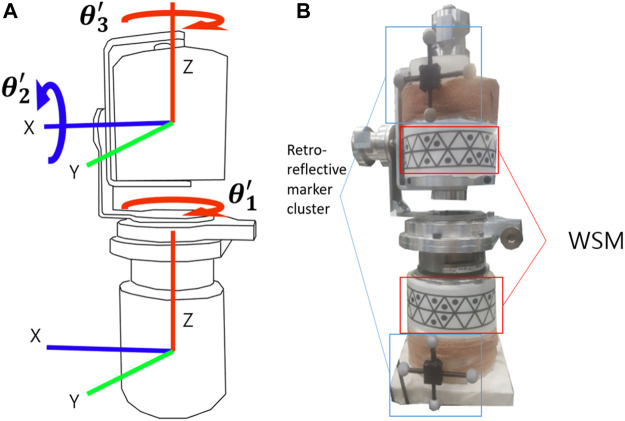
**(A)** Schematic of the gimbal coordinate system. The gimbal had 3 DOFs, and the joint angles were obtained by measuring the position of the upper segment relative to the lower segment. **(B)** Retroreflective cluster and WSM placement on the gimbal. Each cluster and WSM had defined coordinates.

For the gimbal experiment, the gimbal was positioned in the designated motion-analysis area. Retroreflective marker clusters and WSMs were attached to it as shown in Figure 8. With the gimbal encoder output set as the reference, the joint-angle estimation errors for the proposed WSM system and VICON-based system were compared. As shown in Figure 8A, the pose of the upper segment relative to the lower segment of the gimbal was represented by XYZ Euler angles to enable comparison of the estimated joint angles. Additionally, the rotations about the X, Y, and *Z* axes were respectively equated to flexion/extension (Fle/Ext), abduction/adduction (Abd/Add), and rotation (Rot) in this study. The upper part of the gimbal was manually rotated as the joint-angle trajectories captured by the encoders and WSM and VICON-based systems were recorded. Relatively wide and narrow ranges of motion (ROMs) were applied in this experiment. For the narrow ROM test, the gimbal was rotated to maintain a joint-angle ROM equivalent of approximately 5°. For the wide-ROM test, the ROM was adjusted such that the joint-angle equivalent was consistently larger than 10°.

### 3.3 Experiment 3: Joint-Angle Estimation for Lower Limb of a Healthy Human Subject

In the healthy human subject experiment, a modified Helen Hayes marker set methodology was applied for the retroreflective markers (Collins et al., 2009; Charalambous, 2014). Specifically, a total of three retroreflective markers were placed over the sacrum and right and left halves of the anterior superior iliac spine to define the pelvis segment. A total of two markers were placed directly lateral and medial to each knee, and one marker each was placed laterally and medially on the ankle, over the first and fifth digits of the foot, and on the heel of the foot (Figure 1). The marker clusters were also positioned on the thighs and shanks to increase orientation-tracking accuracy. The WSMs were positioned on the pelvis, thighs, and shanks as shown in Figure 1. The predefined anterior and posterior hexagons of each WSM were positioned to be directly anterior and posterior to each segment. The relative camera locations were the same as those for the gimbal experiment, and the subject was asked to walk on a treadmill in the designated motion-analysis area. The XYZ Euler angles obtained by determining the orientation of the distal segment relative to the proximal segment were implemented for joint-angle estimation. For pelvis angle estimation, the coordinates corresponding to the center of the treadmill were set as the proximal segment reference as shown in Figure 7. The joint-angle estimation of the WSM system was compared to the VICON-based system.

The root mean square error (RMSE), standard deviation of difference (SD of Diff), and Pearson correlation coefficient (*r*) were derived for each comparison. Note that a large RMSE does not necessarily indicate a large error, because the different methods used to define the body segment coordinates of the systems introduced a baseline angular offset. According to the paper of Kainz et al. (2017) that compared joint angle estimated from four different clinical marker placing, models using VICON, “PiG-DK”, and “6-DOF-DK” showed about 7° RMSE on hip rotation, and “PiG-DK” and “3-1-1-DOF-IK” showed more than 10° of RMSE in hip flexion/extension. Therefore, RMSE might vary according to the motion-capture method or marker placing protocol. SD of Diff represents the discrepancy between the two systems when the angular offset is ignored. Lastly, the Pearson correlation coefficient indicates the strength of the relationship between the reference and estimated joint trajectories.

To establish a method to assess the accuracy of the proposed WSM system in the presence of skin movement artifacts, we referenced several studies that compared the joint-angle trajectory results obtained as a result of implementing different marker sets in an infrared marker-based system (Ferrari et al., 2008; Collins et al., 2009; Kainz et al., 2017). Ferrari et al. (2008) compared various joint angles estimated for five different retroreflective marker sets; with the exception of the hip rotation and knee abduction/adduction and rotation results that yielded negative correlations, the correlations varied between 0.7 and 1.0. Additionally, Collins et al. (2009) compared the performance of a marker set referred to as the “6-DOF marker set” to that of the Helen Hayes marker set; they found the knee flexion angle to have the largest SD of Diff (3.61°). Kainz et al. (2017) compared four different clinical marker sets, and it showed the largest standard deviation of RMSD of about 5.0° on knee rotation. Their varied results indicate that it is difficult to accurately estimate joint angles if the system is susceptible to skin movement artifacts, because the estimations may vary according to the retroreflective marker set. Thus, we aimed to determine high correlation coefficients and low SD of Diff values that indicated a good result. Specifically, using the results reported by Ferrari et al., Collins et al., and Kainz et al. as references, a correlation coefficient value 0.70 or higher was set as the criterion for a good correlation, whereas an SD of Diff value 5.0° or lower was set as the criterion for a good SD of Diff. These criteria were applied in the analysis of the human experiment results.

Data collection for the human subject was approved by the Institutional Review Board at the Korea Advanced Institute of Science and Technology (KH 2019-113).

## 4 Results

### 4.1 Experiment 1: Robustness of Marker Recognition

We applied the WSM recognition algorithm on the images of the LabelMe dataset, and 27 markers were falsely detected, resulting in a 0.013% (27/207,913 × 100) FP rate. As shown in Table 1, we could observe that the FN rate started to grow as the pixel area of a WSM became smaller than 800 pixels, showing a 0.5% FN rate. When the pixel area became smaller than 500 pixels, FN became 45.3%, indicating that it is hardly usable at the 0–500 pixel area range. As one WSM is composed of 10 triangles, the result indicates that the pixel area of each triangle should be larger than 80 in the image for robust recognition.

**TABLE 1:** FN rate analysis for WSMs.

Pixel area of WSM	3,000∼2,000	2,000∼1,500	1,500∼1,000	1,000∼800	800∼500	500∼400
FN rate (%)	0	0	0	0	0.5	45.3

### 4.2 Experiment 2: Joint-Angle Estimation for a 3-DOF Gimbal

**FIGURE 9:**
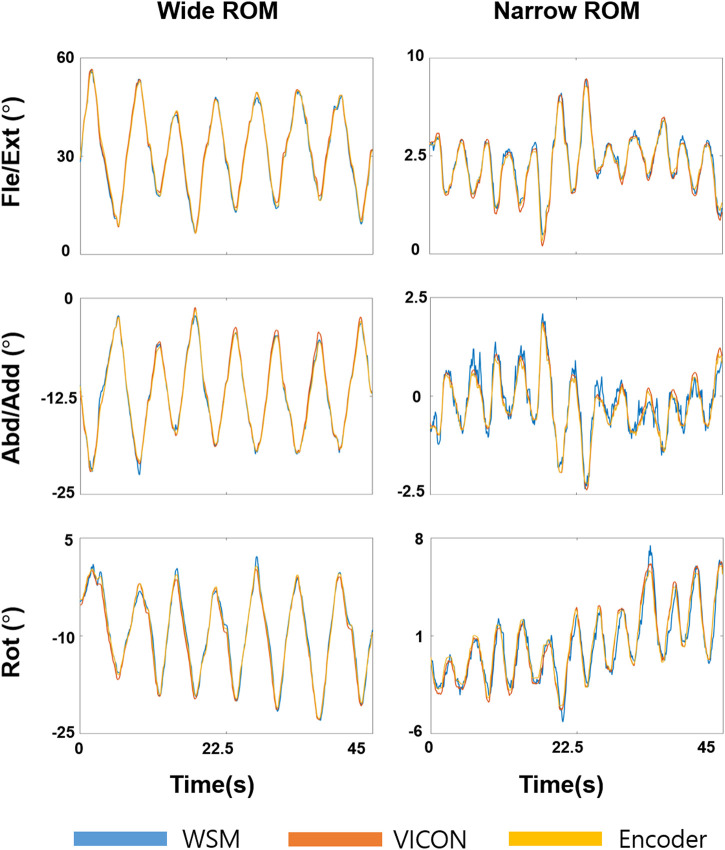
Joint-angle estimation results for the 3-DOF gimbal experiment. Fle/Ext, Abd/Add, and Rot angles were obtained for wide and narrow ROMs. Yellow, orange, and blue respectively denote the encoder, VICON-based system, and WSM system results.

**FIGURE 10:**
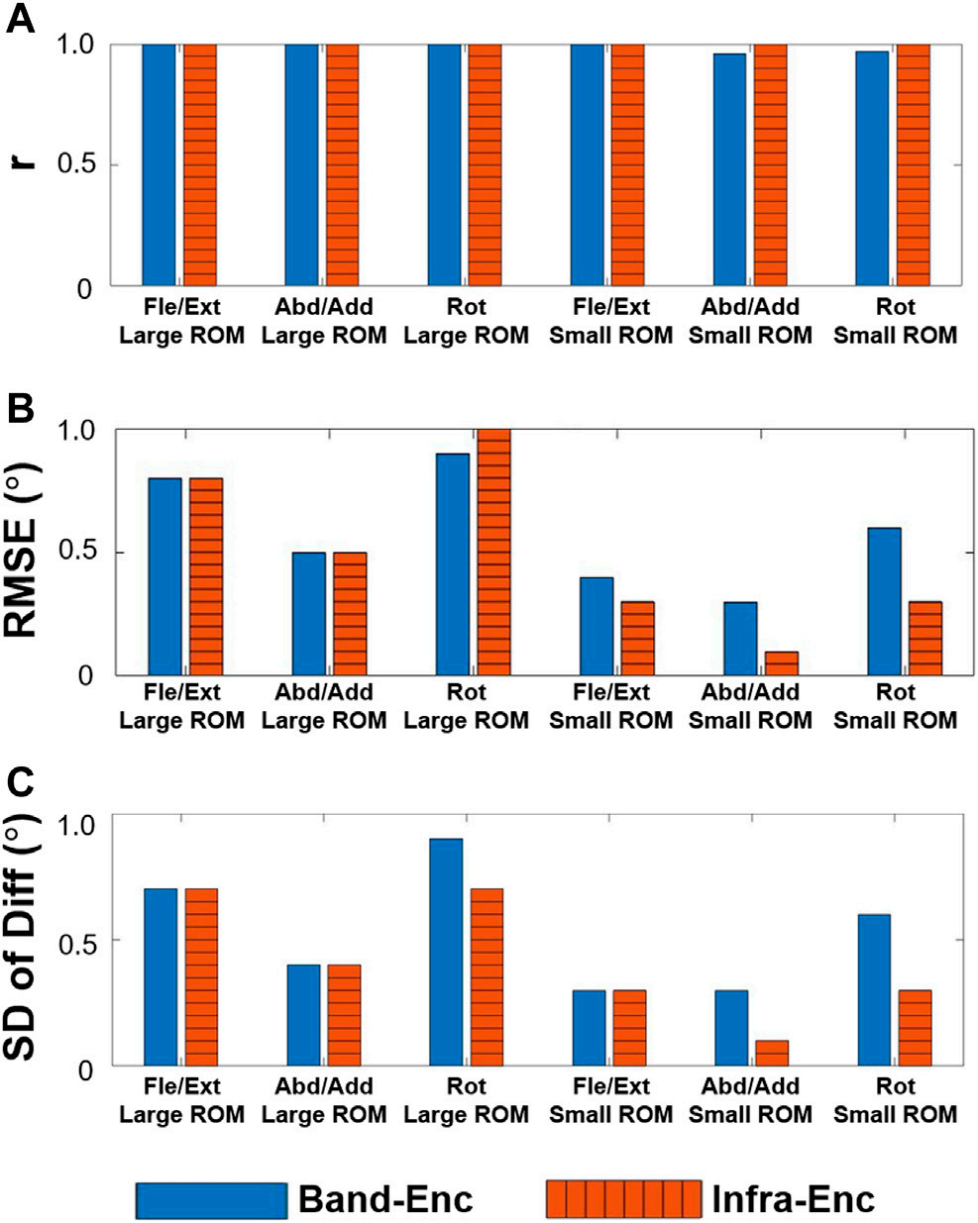
WSM-Enc (blue solid) and Infra-Enc (orange striped) comparison results. High correlation coefficients **(A)** and relatively small discrepancies, as shown by the RMSE **(B)** and SD of Diff **(C)** results, were consistently observed between the two systems.

The results of the gimbal experiment are presented in Figures 9 and 10. The blue solid bars in Figure 10 denote the WSM and encoder system comparison results (WSM-Enc), and the orange striped bars denote the infrared camera-based (VICON-based) and encoder system comparison results (Infra-Enc). It is worth noting that *r* was higher than 0.95, and RMSE and SD of Diff were 1.0° or less for all joint-angle estimations (Figure 10). The WSM-Enc result yielded the largest SD of Diff at Rot with a large ROM, which was 0.9°, while the Infra-Enc result yielded 0.7°. Additionally, even though the Infra-Enc result showed a better or similar SD of Diff result for all joint-angle estimations, the differences of the SD of Diff values between WSM-Enc and Infra-Enc were consistently less than 0.3° (Figure 10C).

### 4.3 Experiment 3: Joint-Angle Estimation for Lower Limb of a Healthy Human Subject

**FIGURE 11:**
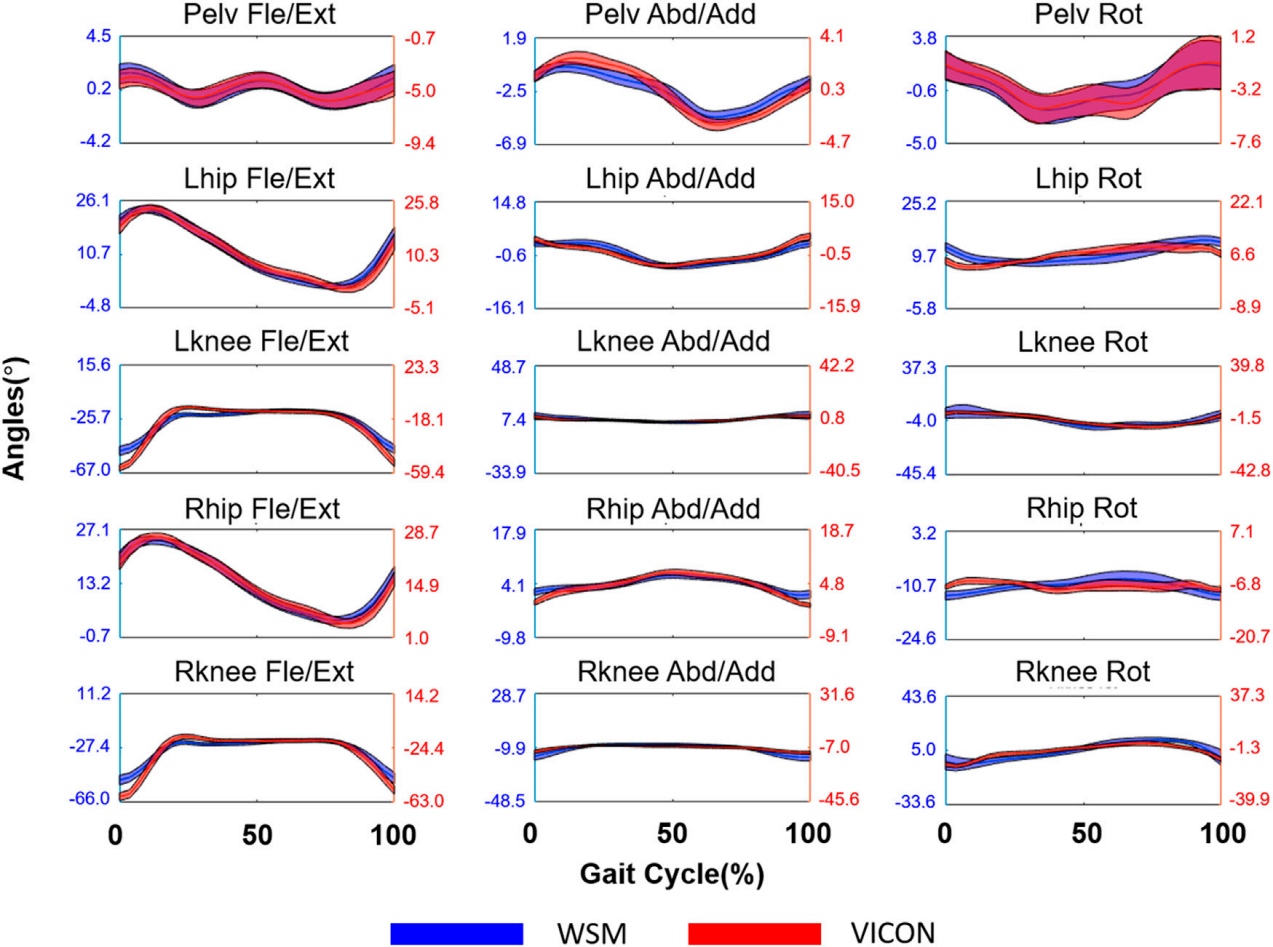
Joint-angle estimation results for the lower limbs of a human subject walking on a treadmill. The blue and red lines respectively denote the WSM and infrared camera-based systems (VICON). The X axis denotes the gait cycle percentage (%), and the *Y* axis denotes the estimated joint angle in degrees (°), which was averaged over all gait cycles.

**FIGURE 12:**
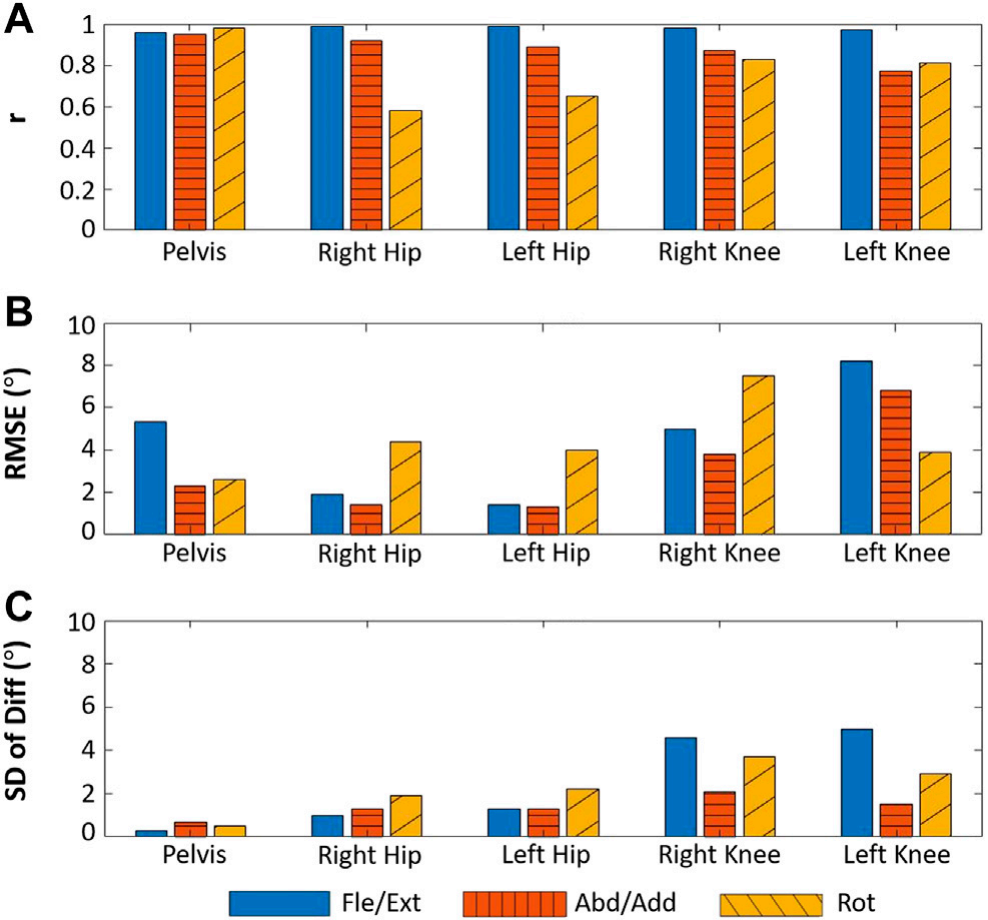
Results for the human subject joint-angle estimation experiment. The blue solid bars denote rotation in the sagittal plane (pelvic tilt, hip Fle/Ext, and knee Fle/Ext), the orange striped bars denote rotation in the frontal plane (pelvic obliquity, hip Abd/Add, and knee Abd/Add), and the yellow diagonally striped bars denote rotation in the transverse plane (pelvic Rot, hip Rot, and knee Rot).

The results of the healthy subject gait analysis experiment are shown in Figures 11 and 12. As shown in Figure 12A, with the exception of the right and left hip rotation results (*r* = 0.58 and 0.65 respectively), *r* was consistently above 0.70; moreover, as can be seen in Figure 12C, all of the SD of Diff values satisfied the criteria for good SD of Diff that we set in section 3.3. The highest SD of Diff for both sides was on Fle/Ext for the knee joints, showing 4.6° and 5.0° on the right and left sides, respectively, but they are still in the range of the criteria for good SD of Diff. RMSE varied from 1.4° to 8.2°, and it might be attributable to the different way of defining coordinates of body segments between the WSM and infrared camera-based systems.

## 5 Discussion

We evaluated the performance of the WSM system through three experiments. Experiment 2 and Experiment 3 showed that the accuracy of the WSM system is comparable to that of the VICON-based system. In Experiment 2, which is the gimbal-based experiment, the difference between the SD of Diff values for the WSM-Enc and Infra-Enc comparisons was consistently below 0.3°. A previous study that focused on quantifying the error associated with the application of a VICON 370 system to estimate the joint angles for a static leg-like object revealed that the error varied between 0.1° and 0.4° owing to changes in the position of the object in the designated motion-analysis area (Dorociak and Cuddeford, 1995). Thus, the error level of the proposed system is within the range of the error for VICON-based systems. Furthermore, the IMU 3-DOF gimbal test reported by Brennan et al. (2011) yielded a 3.19° maximum SD of Diff compared to the potentiometer, whereas the largest WSM-Enc SD of Diff (0.9°) was considerably smaller, indicating that a WSM-based system has the potential to become a general-use motion-analysis system. In Experiment 3, which is the human gait experiment, all of the SD of Diff results satisfied the criteria of high accuracy that we set based on previous studies showing an SD of Diff of 5.0° or less. This result indicates that WSM has a high tracking accuracy comparable to the practically used marker sets of the VICON system.

For the gimbal experiment, the largest WSM-Enc SD of Diff (0.9°) was obtained for the wide-ROM Rot. This may be attributable to coordinate system misalignment. There were three coordinate systems for each segment in the 3-DOF gimbal experiment that were each defined through the use of retroreflective marker clusters, WSMs, and encoders. Ideally, they would have been perfectly aligned for direct comparison; however, because the markers were attached by hand, perfect alignment was not possible. It is for this reason that the Rot SD of Diff increased as the ROM was extended.

Regarding the human subject experimental results, the RMSE values for the proposed and infrared camera-based systems varied between 1.4° and 8.2°. As mentioned in section 3, a large RMSE value does not indicate poor pose estimation performance, as it is an effect of employing different techniques to define the coordinates of the two systems; this resulted in misaligned segment coordinates. Although RMSE was not considered to be an indicator of system performance in this study, the marker placement protocol for the WSM-based system needs to be improved to enable robust definition of segment coordinates. The method used in this study entailed positioning the WSMs such that a specific hexagon center is positioned at the anterior or posterior body segment center. To improve segment coordinate definition, additional guidelines that take into account bony landmarks should be established in future work.

Although all joint-angle estimation satisfied the criterion for good SD of Diff, the knee Fle/Ext showed the largest SD of Diff on both sides. This may have been largely attributable to skin movement artifacts. As previously mentioned, comparisons of the joint-angle results obtained through the use of retroreflective markers and WSMs that cannot be simultaneously positioned on body segments at the same location will be subject to the influence of skin movement artifacts. Gao and Zheng (2008) reported the effects of these artifacts on the thigh and shank to be up to 19.6° and 8.6°, respectively. Their results indicate that differential marker placement can manifest as large deviations in the results. In this study, the hip rotation angle results indicated the lowest correlation (*r* = 0.58); this finding is consistent with the results of previous studies that investigated the implementation of different marker sets in an infrared camera-based system (Ferrari et al., 2008; Collins et al., 2009). Ferrari et al. analyzed the kinematic results obtained through the use of five different marker sets; in some cases, they found negative correlations for hip rotation. Furthermore, in a study performed by Collins et al. that compared the Helen-Hayes set and their “6-DOF marker set”, the results for the hip joint angle in the transverse plane revealed opposing joint-angle trajectory trends, indicating a large discrepancy.

In Experiment 1, we evaluated the robustness of recognition for WSM as it is a newly designed marker. AprilTag and AprilTag2, which are two of the most widely used and robust fiducial markers, were also evaluated on the LabelMe dataset (Wang and Olson, 2016). AprilTag and AprilTag2 respectively showed FP rates of 0.034% (145/421,049 × 100) and 0.0014% (6/421,049 × 100) (the number of images was different from the version that we downloaded). WSM showed an FP rate lower than that of AprilTag but higher than that of AprilTag2, indicating that WSM has comparable robustness to widely used fiducial markers. The reason for an FP higher than that of AprilTag2 might be the deformability of WSM. AprilTag2 is used on planar surfaces, so it can reject the deformed candidates in the sample images, resulting in a low FP rate, while WSM does not reject those candidates as it is designed to be used on curved surfaces. For the FN rate, the result in Table 1 could be the guideline for deciding the size of the motion-capture area and WSM pattern. The size of the WSM pattern in Experiment 2 and Experiment 3 was chosen according to this result.

## 6 Future Study

In this study, we focused on introducing the main concept of the WSM system and evaluating its accuracy in the environment that has a sufficient number of cameras for stable recognition of WSM. Ten low-cost web cameras were placed around a 2.2 m × 2.3 m space. However, the applicability of an optical motion-capture system for home-based use is highly related to the number of cameras and calibration procedure. Therefore, we are planning to evaluate the performance of the WSM system by reducing the number of cameras from ten to two. If the WSM system with a reduced number of cameras still results in acceptable accuracy, it will reduce the system cost, required space, and calibration procedure. Especially with two cameras, it might require only an initial calibration if the relative pose is fixed. Moreover, we will investigate on the light condition and improve the wearing part of WSMs to make the WSM system more suitable for home-based use. The second goal of future studies should be to establish a human experiment protocol that can minimize the effects of skin movement artifacts. This may be achieved by using retroreflective markers with intra-cortical pins or by establishing a protocol based on the 3D bone reconstructions that were obtained via fluoroscopic imaging (Benoit et al., 2006; Zheng et al., 2006; Kurazume et al., 2009). Although the intra-cortical pin method requires surgical preparation and a fluoroscopic imaging system is difficult to apply for continuous joint-angle estimation, these methods may yield the most reliable results for WSM-based systems. Alternatively, methods similar to the point cluster technique and optimal common shape technique have been developed and applied to minimize skin movement artifacts in infrared camera-based systems (Taylor et al., 2005). These techniques take into account the fact that the original marker configuration will change as a subject moves; this discrepancy is quantified and subsequently taken into account in body segment coordinate calculations. This approach can also be applied to WSM pattern vertices to reduce skin movement artifacts. Lastly, a marker placement protocol based on bony landmarks needs to be established for the WSM system.

## 7 Conclusion

A method for affordable optical motion analysis was developed in this study. The use of webcams can reduce the cost of such systems, thereby extending the applicability of optical motion-capture systems to include home-based applications, such as posture correction for at-home training, accurate body tracking for virtual-reality gaming, and remote monitoring for home rehabilitation. WSMs that can be recognized by webcams were designed, and the pose estimation accuracy of the resulting system was evaluated. The results of the human subject experiment revealed a relatively low correlation for left and right hip rotations; we interpreted the main source of error to be skin movement artifacts. Except these results, all of the other joint-angle results met the criteria of high accuracy that we set based on previous studies. The results of the gimbal experiment, which was not subject to skin movement artifacts, revealed the accuracy of the proposed WSM system to be comparable to that of an infrared camera-based system under the condition of encoder output as the reference. The WSM system can be a good solution in the field of home-based motion analysis that requires high accuracy as well as low cost.

## Data Availability

The raw data supporting the conclusion of this article will be made available by the authors, without undue reservation.
